# Large interlayer Dzyaloshinskii-Moriya interactions across Ag-layers

**DOI:** 10.1038/s41467-023-42426-9

**Published:** 2023-10-30

**Authors:** Jon Ander Arregi, Patricia Riego, Andreas Berger, Elena Y. Vedmedenko

**Affiliations:** 1 CIC nanoGUNE BRTA, Tolosa Hiribidea 76, E-20018 Donostia–San Sebastián, Spain; 2 Departamento de Física de la Materia Condensada, Universidad del País Vasco, UPV/EHU, E-48080 Bilbao, Spain; 3 Universität Hamburg, Jungiusstrasse 11a, 20355 Hamburg, Germany

**Keywords:** Magnetic properties and materials, Magnetic properties and materials

## Abstract

Seeking to enhance the strength of the interlayer Dzyaloshinskii-Moriya interaction (IL-DMI) through a combination of atomic and Rashba type spin-orbit coupling (SOC) we studied the strength and the thickness evolution of effective interlayer coupling in Co/Ag/Co trilayers by means of surface sensitive magneto-optical measurements that take advantage of the light penetration depth. Here, we report the observation of oscillatory, thickness-dependent chiral interaction between ferromagnetic layers. Despite the weakness of the Ag atomic SOC, the IL-DMI in our trilayers is orders of magnitude larger than that of known systems using heavy metals as a spacer except of recently reported −0.15 mJ/m^2^ in Co/Pt/Ru(t)/Pt/Co and varies between ≈ ±0.2 mJ/m^2^. In contrast to known multilayers Co/Ag/Co promotes in-plane chirality between magnetic layers. The strength of IL-DMI opens up new routes for design of three-dimensional chiral spin structures combining intra- and interlayer DMI and paves the way for enhancements of the DMI strength.

## Abstract (web summary)

When two magnetic layers are separated by a metallic spacer, an interlayer exchange coupling can occur. Recently, it has been shown that interlayer Dzyaloshinskii-Moriya interactions can also occur, driving chiral spin canting in the magnetic layers. Here, Arregi et al. demonstrate a large interlayer Dzyaloshinskii-Moriya interaction in Co/Ag/Co multilayers, which occurs despite the weak atomic spin-orbit coupling of the Ag spacer layer.

## Introduction

Fundamental research on magnetic interactions at the nanoscale has greatly benefited in the last decades from the increasing ability to control surfaces and interfaces of materials at the sub-nanometer scale. The possibility to grow high-quality multilayer stacks of ultrathin magnetic/nonmagnetic films soon led to the observation of magnetic interlayer exchange coupling (IEC)^1,2^, consecutively followed by the discovery of giant magnetoresistance (GMR)^3,4^, and thus eventually launching the field of spintronics as well as revolutionary advances in magnetic recording technology. The first kind of IEC reported was bilinear in nature, causing parallel or antiparallel alignment of the magnetization vectors in the coupled layers, according to the Heisenberg type energy term $-{J}_{{{{{{\rm{Heis}}}}}}-{{{{{\rm{IEC}}}}}}}{{{{{{\boldsymbol{M}}}}}}}_{1}\cdot {{{{{{\boldsymbol{M}}}}}}}_{2}$, where *J*_Heis–IEC_ is the strength of the isotropic IEC, while ***M***_1,2_ corresponds to the net magnetization of the layers. Detailed successive experiments showed that the sign and strength of *J*_Heis–IEC_ depends on the interlayer thickness in an oscillatory fashion^5^, a fact that was theoretically explained in the framework of RKKY interactions^6^. A second type of interlayer coupling was observed soon thereafter in Fe/Cr/Fe trilayers, promoting a perpendicular magnetization configuration of the magnetic layers^7^. The observation of this non-collinear coupling, termed as biquadratic, was also found in other multilayer systems^8^, and the IEC interaction was generalized by adding a term of the form −*J*_b_(***M***_1_ · ***M***_2_)^2^.

Both bilinear and biquadratic exchange interactions contain a symmetric product of the ***M***_***i***_, such that exchanging them does not influence the energy term. That is, they correspond to the isotropic and/or symmetric parts of the exchange tensor. Opposite to this, the Dzyaloshinskii–Moriya interaction (DMI)^9,10^, expressed as −***D*** · (***M***_1_ × ***M***_2_), represents an antisymmetric part of the exchange tensor and favors an orthogonal orientation of ***M***_1_ and ***M***_2_, generating configurations with a specific helicity defined by the coupling vector ***D***. Despite this type of interaction only being allowed in systems with broken inversion symmetry, a seminal work by A. Fert suggested that a nonvanishing DM interaction can appear in the context of ultrathin magnetic films under the presence of spin–orbit coupling (SOC), due to the reduction of symmetry at interfaces^11^. This so-called interfacial DMI has recently attracted vast interest, resulting in further theoretical and experimental works focused on the rich variety of non-trivial spin configurations arising from it, including spin spirals, chiral domain walls, and skyrmions^12–21^. Hereby, it is worthwhile to notice that all such textures are intralayer magnetic configurations because the standard interfacial DMI defines an intralayer coupling only. In order to enhance DMI and stabilize intralayer skyrmions for the purpose of utilizing them as bits of information, for instance, multilayers of magnetic and nonmagnetic metals with multiple interfaces have been proposed^21^. These multilayers typically show collective behavior; that is, spin configurations in all layers are identical due to a strong ferromagnetic interlayer exchange and can be effectively regarded as one single layer with intralayer DMI.

Recent investigations, however, revealed several systems possessing sizable interlayer DMI (IL-DMI) acting between magnetic layers across a non-magnetic spacer layer^22–27^. In these studies, a chiral bias leading to a direction-dependent hysteresis loop shift by approximately 10^−3^ Tesla was observed and unambiguously attributed to the chiral IL-DMI (0.005–0.01 mJ/m^2^). Simultaneously, a theoretical investigation^28^ explained why this kind of chiral interlayer coupling^29^ was elusive for many years, given that a certain degree of magnetic or structural inhomogeneity within the magnetic layers is needed to facilitate a net IL-DMI^28^.

For any kind of DMI to exist, including IL-DMI, two key ingredients are essential: breaking of inversion symmetry and a strong SOC. While symmetry breaking in multilayers is ensured by the interfaces, strong SOC is typically provided by the electronic properties of heavy metals with unfilled d-shells (Pt, Ir, or Pd) used as spacing layers, and so far, all investigations on IL-DMI were limited to this sample design^22,24–26^. It is, however, known that lighter materials with very weak intrinsic spin–orbit parameters can nevertheless develop strong SOC due to the large Rashba splitting of their (non-polarized) band structure^30–32^. It is unclear so far whether the combination of Rashba- and atomic SOC can be used to strongly enhance or induce the IL**-**DMI, making it competitive with its intralayer counterpart and, by that means to manipulate characteristics of the IL**-**DMI. Hence, it is crucial to explore this possibility in order to significantly broaden the class of materials that can be utilized for the creation of three-dimensional topological systems, which are very relevant for future applications.

In this work, we experimentally explore polycrystalline Co/Ag/Co stacks grown by magnetron sputtering. Hereby, Co belongs to the class of ferromagnets with dominating direct Heisenberg exchange interaction in the range of 0.7–2 × 10^−11^ J/m (0.86–6 meV/bond)^33^, while Co/Ag/Co multilayers have been reported to only exhibit very weak antiferromagnetic RKKY interlayer exchange interactions (~−0.014 mJ/m^2^ ^34^). While Ag is also known for its large Rashba SOC (ξ_Ag_Rashba_ ~ 0.11 eV^35^), we were not able to find any data on interfacial DMI effects in Co/Ag superlattices. Here, we report the experimental observation of a significant IL-DMI between the ferromagnetic Co layers. By using the capabilities of magneto-optics to resolve the magnetization vector during reversal and to distinguish coherent and non-coherent magnetization rotation processes, we evidence that the topmost Co layer features a coherent rotation process with a repeatable helicity that is Ag spacer thickness dependent. In contrast to known IL-DMI systems showing magnetization rotation in reference to the surface normal, our Co/Ag/Co trilayers show an in-plane rotation of magnetization in between the Co layers. By mapping this Ag thickness-dependent rotation to an effective macroscopic coupling of the form −***D*** · (***M***_1_ × ***M***_2_), we estimate the strength of the IL-DMI to be of the order of several 0.1 mJ/m^2^. Because Ag possesses negligible atomic SOC (0.01 eV^35^), but instead a significant energy shift due to Rashba band splitting at the Co/Ag interface^36^, we attribute the emerging IL-DMI to the interplay of inversion symmetry breaking and strong Rashba-induced SOC enhanced by the three-site Lévy-Fert mechanism due to Co impurities within the Ag host. The unusual in-plane chirality is furthermore associated with magnetic inhomogeneities within the Co layers that are related to their polycrystalline structure^28^.

## Results

**Fig. 1:**
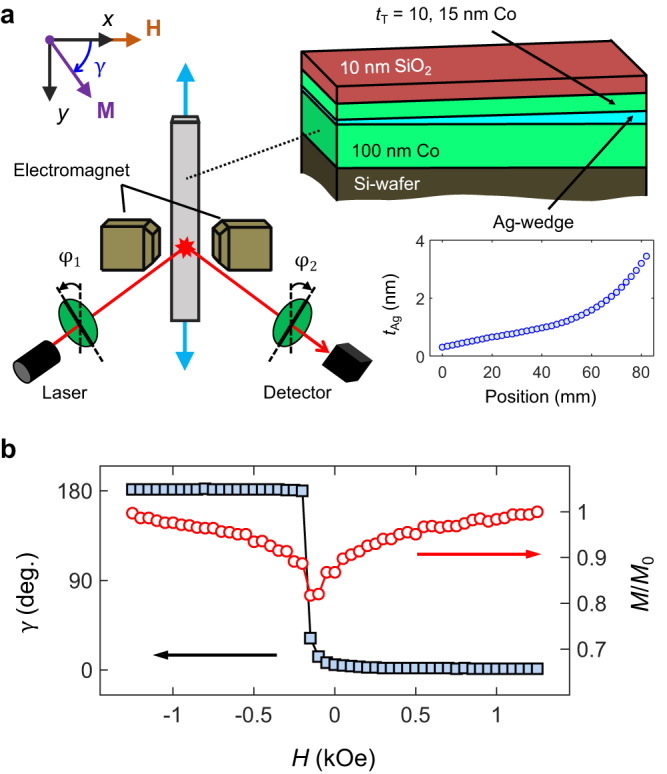
Experimental set-up. **a** Schematic of the sample structure and experimental set-up. The inset contains the Ag-interlayer thickness profile, as well as the definition of the reference frame, field *H* axis, and magnetization orientation *γ*. **b** Field dependence of *γ*, *M*/*M*_0_ for the *t*_*T*_ = 10 nm sample at *t*_Ag_ = 0.77 nm. Typical (median) error bar values for *γ*, *M*/*M*_0_ are 0.3° and 0.01, respectively.

The multilayers in our study were sputter deposited at room temperature onto Si substrates of elongated shape, 80 mm × 5 mm in size. The deposition of the Co layers was carried out by rotating the substrate holder in order to obtain thickness uniformity. In contrast, the substrate was aligned with its long axis toward the direction of a tilted sputter gun for depositing the Ag interlayer so that a position-dependent Ag-thickness could be obtained. Thus, our samples possess a bottom 100-nm-thick Co layer, an Ag-wedge with a thickness ranging from 0.3 to 3.5 nm, and a topmost Co layer of thickness *t*_*T*_ = 10 or 15 nm. This thickness range permits us to achieve the best possible top Co-layer signal isolation due to the magneto-optical depth sensitivity in the generalized magneto-optical ellipsometry (GME) measurements. The large thickness of the bottom layer ensures stable orientation of its net magnetization. An additional 10-nm-thick SiO_2_ overcoat of uniform thickness was subsequently deposited in order to prevent oxidation. Figure 1a shows a schematic of the fabricated sample, with the thickness profile of the Ag-wedge displayed in the inset. The film thicknesses and the wedge profile were calibrated via X-ray reflectivity and spectroscopic ellipsometry. The 2-nm-thick native oxide on Si is sufficient to disrupt any textured growth of the bottom Co layer^37^ so that the resulting Co/Ag/Co films are polycrystalline.

Magnetization reversal of our samples was characterized by means of the magneto-optical Kerr effect (MOKE), using the setup shown in Fig. 1a. The magneto-optical probe was scanned along the wedge’s long axis (*y*-axis) in order to evaluate the effect of the interlayer thickness onto the magnetization behavior of the Co/Ag/Co stack in a quasi-continuous fashion. The setup consists of a laser light source (λ = 635 nm) with a spot size of ~1 mm that illuminates the sample at a 45° angle of incidence, as well as a pair of rotatable linear polarizers selecting the polarization state of light incident on the sample and the photodetector (represented by *φ*_1_ and *φ*_2_, respectively). An electromagnet provides applied magnetic fields up to 1.3 kOe along the *x*-axis, orthogonal to the wedge’s long axis, and aligned within the sample plane. Polarization detection was based on the GME technique^38,39^, allowing for the determination of the complete reflection matrix *R* of the sample. For in-plane magnetized materials,

1 $$R=\left(\begin{array}{cc}{r}_{{ss}} & {r}_{{sp}}\\ {r}_{{ps}} & {r}_{{pp}}\end{array}\right)=\left(\begin{array}{cc}{r}_{s} & \alpha \\ -\alpha & {r}_{p}+\beta \end{array}\right)={r}_{p}\left(\begin{array}{cc}{\widetilde{r}}_{s} & \widetilde{\alpha }\\ -\widetilde{\alpha } & 1+\widetilde{\beta }\end{array}\right),$$

encompassing the purely optical Fresnel reflectivity ${\widetilde{r}}_{s}$, as well as the magnetically induced matrix elements $\widetilde{\alpha }$ and $\widetilde{\beta }$, associated with the longitudinal and transverse MOKE, respectively. The consideration of in-plane magnetization components only is justified here by the thin-film character of the sample in conjunction with the in-plane applied field orientation. The procedure for the reflection matrix measurement consists now in extracting the fractional intensity change $\delta I/I=2\left[I\left(H\right)-I\left(-H\right)\right]/[I\left(H\right)+I(-H)]$ for every field value upon magnetization reversal for a sufficiently large number of (*φ*_1_, *φ*_2_) orientation pairs^38–41^. Subsequently, one fits the known analytical *δI*/*I* (*φ*_1_, *φ*_2_, *R*) expression with the reflection matrix *R* elements as adjustable parameters, obtaining their evolution vs. *H*^42^. An important element of the GME measurement strategy is the fact that optical, magneto-optical, and magnetization orientation information can be adequately separated^42^, furthermore allowing one to recover the dielectric tensor of a sample by subsequently fitting the data to an optical layer model^38–41,43^. This permits us to extract magnetization vector information from our data, which we then utilize to distinguish coherent and non-coherent magnetization rotation processes, with the latter one being indicative of non-uniform magnetization states within each Co layer.

Figure 1b displays the field dependence of the in-plane magnetization orientation angle *γ* (with respect to the field axis) and modulus *M*/*M*_0_ for the sample with a top Co layer of *t*_*T*_ = 10 nm, measured at the position where the interlayer thickness *t*_*Ag*_ = 0.77 nm. Due to the skin depth of light, our experiment is primarily sensitive to the topmost Co layer, and thus we base our initial data analysis here on a semi-infinite top Co-layer in order to obtain the quantities *γ* and *M*/*M*_0_^39^. The *γ* vs. *H* data in Fig. 1b indicate that the topmost Co layer magnetization points along the positive *x*-axis (*γ* ~ 0°) for *H* > 0.9 kOe, thus being well aligned with the field axis. The *M*/*M*_0_ quantity acquires a value close to unity at these applied fields, suggesting a nearly uniform magnetization state. Conversely, at zero field, *M*/*M*_0_ reduces to ~0.85, while *γ* is approximately 6°. The reduction of *M*/*M*_0_ in remanence is the result of non-coherent rotation processes of laterally varying magnetization vectors within the grains that form the polycrystalline Co film due to the distribution of easy axes^37^. Furthermore, the departure of *γ* from zero in remanence indicates that a transverse magnetization also occurs during reversal, which implies the existence of an additional coherent rotation and specifically a preferred clockwise helicity for the magnetization reversal path (see the definition of *γ* in Fig. 1a) upon removing the field. Such a coherent rotation process is absent in all single-layer polycrystalline Co films we prepared, which all possess random in-plane distributions of easy axes^41^.

**Fig. 2:**
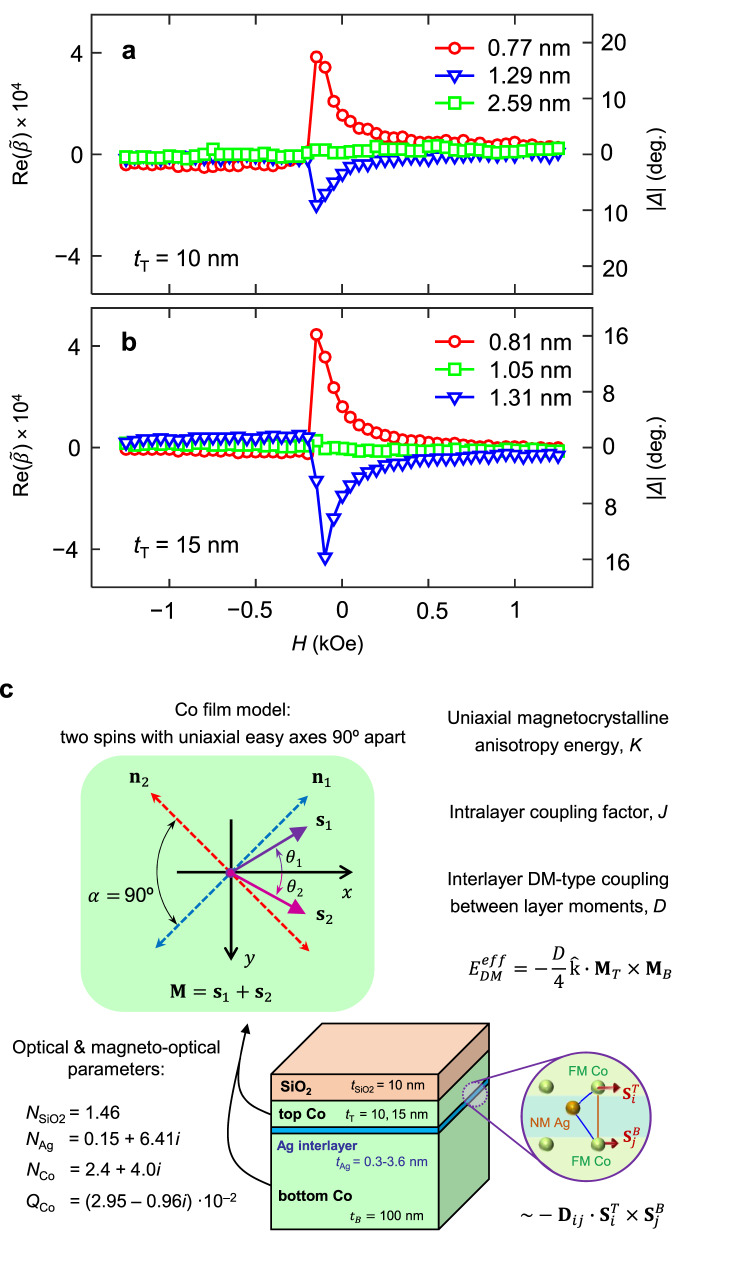
Field-dependence of the transversal magnetization for different Ag thicknesses. **a**, **b** Field dependence of the measured reflection matrix element ${{{{\mathrm{Re}}}}}(\widetilde{\beta })$, proportional to the transverse magnetization component *m*_*y*_, for different Ag interlayer thicknesses in the **a** *t*_*T*_ = 10 nm and **b** 15 nm samples. The right axis of the plots shows the deviation angle |*Δ*| of the top Co layer magnetization from the *x*-axis, assuming sin *γ* ~ *γ* for small angles, illustrating the size of the coherent rotation process. **c** Phenomenological model. Schematic of the combined optical and magnetic model, including all model parameters (see manuscript text).

We investigated the appearance of this coherent magnetization rotation process for different interlayer thicknesses of our Co/Ag/Co samples. Figure 2a, b exhibits ${{{{\mathrm{Re}}}}}(\widetilde{\beta })$ (∝ *m*_*y*_) vs. *H* data measured at three different interlayer thickness positions for the *t*_*T*_ = 10 nm and the 15 nm sample. For *t*_*T*_ = 10 nm, the reversal of the topmost Co layer occurs with a coherent clockwise, anticlockwise, and purely non-coherent rotation for *t*_Ag_ values of 0.77, 1.29, and 2.59 nm, respectively (Fig. 2a). A similar behavior is found in the *t*_*T*_ = 15 nm case, where coherent clockwise and anticlockwise rotations were detected for *t*_Ag_ values of 0.81 and 1.31 nm (Fig. 2b), respectively. In contrast, no coherent rotation was observed at the intermediate value of *t*_Ag_ = 1.05 nm, suggesting that a crossover between preferred clockwise and anticlockwise helicities exists. Moreover, any indication of a coherent rotation process during reversal disappears for high enough interlayer thicknesses (*t*_Ag_ > 2.5 nm) in both samples. At this point, it is worth noting that the appearance of a transverse magnetization *m*_*y*_ here cannot be caused by the stochastic nature of magnetization reversal, given that the extracted ${{{{\mathrm{Re}}}}}(\widetilde{\beta })$ data are the results of hundreds of reversal events measured independently within each GME experiment^41^. A non-zero ${{{{\mathrm{Re}}}}}(\widetilde{\beta })$ value hence means that we see a reproducible and *t*_Ag_-dependent helicity of the topmost Co layer magnetization during reversal. The corresponding magnetization rotation angles |∆| away from the field direction are extracted from our GME data and can be seen by looking at the right-hand axis labels of Fig. 2a, b.

The fact that *m*_*y*_ is always zero for a thick enough interlayer strongly suggests that the observed behavior originates from an effective exchange interaction mechanism between the two ferromagnetic Co layers across the nonmagnetic Ag spacer. However, a magnetization configuration with a predefined helicity cannot be explained in terms of bilinear or biquadratic coupling mechanisms alone since none of those would favor a well-defined clockwise or anticlockwise coherent rotation, such as the one observed here^41^. A hypothetical variation of local magnetocrystalline anisotropy distributions upon changing the spacer layer thickness is also extremely unlikely, given the non-monotonic *t*_*Ag*_ dependence and its disappearance above 2.5 nm thickness. Alternatively, an antisymmetric exchange coupling such as the IL-DMI provides the specific mechanism that would explain the predefined helicity upon reversal that we observe in our Co/Ag/Co samples. Therefore, in the following, we consider an effective IEC dominated by the IL-DMI contributions.

## Discussion

Recent investigations show that an effective interlayer coupling based on the DMI can emerge in ferromagnet/nonmagnetic heavy metal/ferromagnet trilayers^24–27^. In all these investigations, the role of heavy metal was assumed by Pt, which has a strong atomic SOC of 0.51 eV^44^. The atomic SOC of Ag, however, is 0.01 eV only^35^ and, hence, cannot explain the sizable IL-DMI in our samples. On the other hand, the Ag/Co interface shows significant energy shifts due to Rashba band splitting^35,36^. While data in the literature on SOC due to the Rashba effect at Ag/Co interfaces are limited, Ag is generally known to form different metal/Co interfaces or alloys with strong Rashba splitting^45^. Hence, the SOC required for the existence of IL-DMI observed in our experiments might come from the Rashba energy shift at the Co/Ag interface.

Another possibility to obtain non-negligible SOC in Co/Ag/Co stacks is the three-site Lévy–Fert mechanism of DMI^12,44^ with Co impurities within the Ag host serving as mediating sites (atomic SOC of Co is 0.065 eV^44^). Usually, the three-site mechanism requires magnetization rotation in the XZ plane of a stack (like in^24,25^), while an in-plane XY magnetization rotation was observed in our experiments. However, the in-plane helicity can be explained by recent results^28^. In^28^, it has been shown theoretically that the presence of out-of-plane magnetic non-collinearities within ferromagnetic layers can cause a relative in-plane rotation of net magnetizations in between the layers in the framework of the Lévy–Fert model^28^. The rotation chirality is determined by the relative phase of the out-of-plane modulations, which might be defined by sample preparation or other boundary conditions. Most importantly, though, the presence of non-uniform magnetization states within each layer is crucial for the interaction to build up to the macroscopic scale^44^. Here, it is the polycrystalline nature of our Co/Ag/Co samples that straightforwardly accomplishes the nonhomogeneous magnetization scenario within each layer as the necessary condition for macroscopic IL-DMI with in-plane chirality.

A complete discrimination between these two scenarios requires extensive theoretical investigations and, thus, goes beyond the scope of this paper. However, in order to quantitatively map our experimental data onto the DM coupling described above, we have devised a combined optical and magnetic model, through which we attempt to mimic the magnetization reversal behavior of our samples at different *t*_Ag_ values by direct comparison to the extensive data sets that we have acquired.

The key ingredients of our phenomenological model are presented in Fig. 2c, where each ferromagnetic Co layer is represented by two macroscopic spins with anisotropy energy density *K* and misaligned uniaxial anisotropy axes to mimic magnetization modulation within the layers. Additionally, effective intralayer ferromagnetic coupling between spins is also considered by introducing the volume energy density *J*. Finally, an IEC including antisymmetric IL-DMI of the form −*J*_Heis–IEC_ ***M***_1_ · ***M***_2_ − ***D*** · (***M***_1_ × ***M***_2_) + *J*_b_ (***M***_1_ · ***M***_2_)^2^ couples both Co layers, for which we have chosen an effective interlayer ${{{{{\boldsymbol{D}}}}}}{{{{{\boldsymbol{=}}}}}}D\hat{k}$ vector oriented along the vertical axis. An appropriate multilayered optical model of the Co/Ag/Co stacks was also considered (see Fig. 2c), such that the combined outcome of the optical and magnetic model can be directly compared to the experimentally determined reflection matrix elements vs. *H* and *t*_Ag_. Additional details of this combined model can be found elsewhere^38^.

**Fig. 3:**
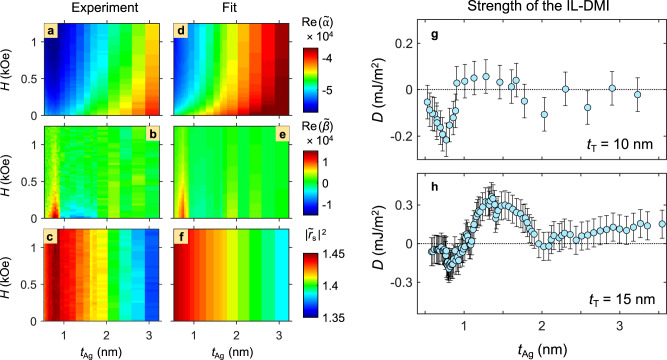
Reflection matrix maps and IL-DMI strength. Color-coded maps of the experimentally determined reflection matrix quantities **a** ${{{{\mathrm{Re}}}}}(\widetilde{\alpha })$, **b** ${{{{\mathrm{Re}}}}}(\widetilde{\beta })$, and **c** ${\left|{\widetilde{r}}_{s}\right|}^{2}$ vs. *t*_*Ag*_ and *H* for the sample with *t*_*T*_ = 10 nm. **d**–**f** The side-by-side corresponding model fits according to the combined optical and magnetic model; **g**, **h** Thickness dependence of the interlayer DMI. *t*_Ag_ dependence of the DM type interlayer coupling strength *D*, as obtained from the fit to the combined optical and magnetic model of the multilayer system, for the sample with **g** *t*_*T*_ = 10 nm, **h** 15 nm. The error bars in (**g**) and (**h**) correspond to the standard deviation of the experimental data.

Color-coded maps of the experimentally determined reflection matrix elements ${{{{\mathrm{Re}}}}}(\widetilde{\alpha })$, ${{{{\mathrm{Re}}}}}(\widetilde{\beta })$, and ${\left|{\widetilde{r}}_{s}\right|}^{2}$ vs. *t*_Ag_ and *H* are shown in Fig. 3a–c for the *t*_*T*_ = 10 nm sample. Especially interesting is the behavior of the parameter ${{{{\mathrm{Re}}}}}(\widetilde{\beta })$ in Fig. 3b, proportional to the transverse MOKE of the sample. For large applied fields, its value is nearly zero for all Ag thicknesses. However, as the field is reduced towards *H* = 0 Oe, an oscillatory behavior emerges, as ${{{{\mathrm{Re}}}}}(\widetilde{\beta })$ features sign changes as well as an attenuation of its amplitude down to zero with increasing *t*_*Ag*_. The longitudinal MOKE parameter ${{{{\mathrm{Re}}}}}(\widetilde{\alpha })$ (Fig. 3a) and the optical reflectivity ${\left|{\widetilde{r}}_{s}\right|}^{2}$ (Fig. 3c) feature a reduction of their amplitude as *t*_Ag_ increases, given that a thicker Ag interlayer reduces the amount of MOKE signal coming from the bottom Co layer as well as changes the optical reflectivity of the Co/Ag/Co stack, respectively. In addition, ${{{{\mathrm{Re}}}}}(\widetilde{\alpha })$ also shows a decrease of its amplitude upon lowering the field, which reflects primarily the non-coherent magnetization rotation process described above.

We have simultaneously fitted the data in Fig. 3a–c to the combined optical and magnetic model presented in Fig. 2c by adjusting the DM coupling strength ***D***, *J*_Heis–IEC_, and *J*_b_ for each Ag thickness value while keeping all other material parameters constant for all *t*_Ag_ as shown in METHODS. The values of *J*_b_ and *J*_Heis-IEC_ appear to be an order of magnitude weaker than that of ***D***. Additionally, both fit curves using all three coupling parameters (***D***, *J*_Heis–IEC_ and *J*_b_), and the ***D*** parameter only lie within the standard deviation margins of the experiment (see^41^), so that we cannot reliably distinguish between them. However, the data cannot be explained in the absence of ***D***. Therefore, we have limited the analysis to the determination of the effective chiral contribution.

The fitted quantities are presented side-by-side with the measured ones in Fig. 3d–f, displaying a very good quantitative agreement. The same level of excellent agreement is also observed for the *t*_*T*_ = 15 nm sample^41^. The *t*_Ag_ dependence of the fitted DM type coupling strength is shown in Fig. 3g, h for both Co/Ag/Co samples. There is an Ag onset thickness of around ~0.6 nm, at which the coupling factor *D* first departs from zero. It then changes its sign in an oscillatory fashion for both samples with different top Co layer thicknesses while attenuating towards the thick end of the interlayer, confirming that the coupling between the Co layers should disappear for a sufficiently thick non-magnetic spacer. We find that the strength of IL-DMI is of the order of ~ 0.1 mJ/m^2^, which is sufficient to generate rather large magnetization loop modifications in our samples, requiring field strengths of up to nearly 100 mT to suppress the IL-DMI effect and align the magnetization vectors in our bilayer samples. The IL-DMI strength does not show any significant in-plane azimuthal angle dependence, as explained in Fig. S5 of the supplementary material. The reasons are the polycrystalline structure of our samples with a random distribution of in-plane anisotropy axes due to our fabrication procedures as well as the resulting normal orientation of the effective IL-DMI vector.

Hereby it is worthwhile to notice that prior experimental reports of IL-DMI^24–26^ led to magnetic state modifications exhibiting field effects of only about 1 mT, even though the magnetic films in those studies were about an order of magnitude thinner than the 10–15 nm top layer films we used in our work here, and thus should have been impacted more strongly by an interface effect^46^. While the Co thickness in our experiments is larger than that in previous investigations, we do not find the results for the 10 nm and 15 nm top Co layers to be very different from each other. Indeed, they both show oscillatory behavior that is nearly in phase, meaning that the zero crossings of the IL-DMI are very similar and the coupling sign identical. Also, the size of the effect is very comparable. The observed rotation angles in Fig. 2 are smaller for the thicker Co-film, at least for the first peak at around 0.8 nm. This effect, however, does not seem to show a simple interface term thickness dependency. One possible explanation for the enhancement with respect to thinner Co films IL-DMI is an increasing Rashba energy shift in a certain thickness range of magnetic metals^47^. Another explanation is the non-trivial thickness dependence of IL-DMI that can, up to a certain point, increase with the thickness, as shown in Fig. 3 of ref. ^24^. The microscopic reason for such a behavior is the cumulative electron hopping corresponding to the fact that with increasing thickness of the magnetic layer, the electrons can scatter not only with the nonmagnetic atoms of a spacer layer (Ag in our case) but also with magnetic atoms (Co in our case).

The SOC due to the Rashba effect at the Ag interfaces is an order of magnitude larger than the atomic SOCs of Ag and Co: ξ_Ag_Rashba_ ~ 0.11 eV^35^. The strength of the DMI is linearly proportional to the SOC in the heavy-metal systems as well in the Rashba systems^48^. The Rashba SOC decreases only very slowly with the layer thickness as shown, e.g., in Fig. 4b of ref. ^35^ (for Ag 5p, it decreases from 0.11 eV to 0.08 eV over 50 nm of Ag thickness). Hence, for our Ag thickness below 3 nm, it has its maximal value. Therefore, in our understanding, the total action of the atomic and Rashba SOCs can lead to the much higher IL-DMI values that we observe here than what was reported in previous investigations. Furthermore, it is important to mention that our experimental observations here are in good agreement with the predictions of ref. ^28^ and also exhibit a previously undetected oscillatory behavior. The maximal strength of IL-DMI is approximately one order of magnitude lower than the intralayer DM interaction found in bilayer and trilayer stacks with perpendicular magnetic anisotropy^49,50^.

In conclusion, we have shown the existence of a coherent magnetization rotation process with predefined helicity in the top ferromagnetic layer of polycrystalline Co/Ag/Co stacks via detailed MOKE measurements. We argued that the observed behavior unambiguously indicates the existence of an interlayer DMI. The strength of discovered IL-DMI is orders of magnitude stronger than that in known systems and promotes a scissor-like in-plane state between the magnetization vectors of the two Co layers that follows a predefined chirality. The interaction appears due to the combination of inversion symmetry breaking with strong SOC, arising either from the strong Rashba SOC at Co/Ag interfaces or from the Lévy–Fert three-site coupling via Co impurities in the Ag interlayer matrix, or from the interplay of both mechanisms. Importantly, the atomic scale interaction can build up to an effective interlayer coupling if non-uniform magnetization states are present. Additionally, the Co/Ag/Co stacking here is the first system showing in-plane chiral rotation of magnetic layers. We have also found that the sign and strength of the interaction varies in an oscillatory fashion with the spacer thickness, attenuating considerably for values above 2 nm. The type of interlayer coupling that we have found here could lead to field-free in-plane spin–orbit–torque switching of the top layer, strong chiral GMR effects, and other new strategies for the generation and manipulation of chiral spin structures in multilayers and broaden the class of materials showing IL-DMI to lighter nonmagnetic metals with weak atomic SOC. Further studies on different materials as well as attempts for controlled placement of nonmagnetic impurities in the spacer will allow for optimization and tuning of the interaction.

## Methods

### Generalized magneto-optical ellipsometry (GME) methodology

#### Reflection matrix determination procedure

The GME technique allows the determination of the full reflection matrix of the sample (see Eq. (M1) in the manuscript text), the maximum information that can be obtained within an optical reflection experiment. The advantage of the conceptually simple setup employed here is that the electric field of light reaching the photodetector can be written as *E*_*D*_ = *P*2 · *R* · *P*1 · *E*_*I*_, where *E*_*I*_ is the electric field of the incident light, *P*1 and *P*2 correspond to the Jones matrices of the linear polarizers in the setup (dependent on *φ*_1_ and *φ*_2_, respectively), and *R* is the reflection matrix of the sample for a given magnetization state. The corresponding light intensity function measured at the photodetector is then written as $I={E}_{D}^{*}\cdot {E}_{D}$. By recalling the time-reversal symmetry ***M***(***H***) = −***M***(−***H***) for ferromagnets and assuming that the magnetically induced matrix elements $\widetilde{\alpha }$ and $\widetilde{\beta }$ change the sign under inverting *H*, one can express the fractional intensity change at a given field *H* upon magnetization reversal as^38,39^

M.1 $$\frac{\delta I}{I}\left({\varphi }_{1},\, {\varphi }_{2}\right)=\frac{I\left(+H\right)-I(-H)}{\left[I\left(+H\right)+I\left(-H\right)\right]/2}=4\frac{{B}_{1}\,{f}_{1}+{B}_{2}\,{f}_{2}+{B}_{3}\,{f}_{3}+{B}_{4}\,{f}_{4}}{{f}_{3}+{B}_{5}\,{f}_{5}+{2B}_{6}\,{f}_{4}}$$

with the six real *B*_*i*_ parameters being related to the three complex numbers ${\widetilde{r}}_{s}$, $\widetilde{\alpha }$ and $\widetilde{\beta }$ as

M.2 $$\begin{array}{cc}{B}_{1}={{{{\mathrm{Re}}}}}(\widetilde{\alpha }) & {B}_{2}={{{{\mathrm{Re}}}}}({\widetilde{r}}_{s}\cdot {\widetilde{\alpha }}^{*})\\ {B}_{3}={{{{\mathrm{Re}}}}}(\widetilde{\beta }) & {B}_{4}={{{{\mathrm{Re}}}}}({\widetilde{r}}_{s}\cdot {\widetilde{\beta }}^{*})\\ {B}_{5}={\left|{\widetilde{r}}_{s}\right|}^{2} & {B}_{6}={{{{\mathrm{Re}}}}}({\widetilde{r}}_{s})\hfill\end{array}$$

On the other hand, the *f*_*i*_ are trigonometric functions of the polarizer angles *φ*_1_ and *φ*_2_

M.3 $${f}_{1}\left({\varphi }_{1},\, {\varphi }_{2}\right)	={\sin }^{2}{\varphi }_{1}\sin {\varphi }_{2}\cos {\varphi }_{2}-{\sin }^{2}{\varphi }_{2}\sin {\varphi }_{1}\cos {\varphi }_{1}\\ {f}_{2}\left({\varphi }_{1},\, {\varphi }_{2}\right)	={\cos }^{2}{\varphi }_{2}\sin {\varphi }_{1}\cos {\varphi }_{1}-{\cos }^{2}{\varphi }_{1}\sin {\varphi }_{2}\cos {\varphi }_{2}\\ {f}_{3}\left({\varphi }_{1},\, {\varphi }_{2}\right)	={\sin }^{2}{\varphi }_{1}{\sin }^{2}{\varphi }_{2}\\ {f}_{4}\left({\varphi }_{1},\, {\varphi }_{2}\right)	=\sin {\varphi }_{1}\cos {\varphi }_{1}\sin {\varphi }_{2}\cos {\varphi }_{2}\\ {f}_{5}\left({\varphi }_{1},\, {\varphi }_{2}\right)	={{\cos }^{2}{\varphi }_{1}\cos }^{2}{\varphi }_{2}$$

Formally, performing six reflection experiments at different (*φ*_1_, *φ*_2_) polarizer orientations is enough to characterize the six real *B*_*i*_ parameters defining the reduced reflection matrix $\widetilde{R}$. However, we proceed by collecting a relevant number of *δI*/*I* data for different (*φ*_1_, *φ*_2_) configurations near the crossing point, at which the polarizer axes become perpendicular and *δI*/*I* exhibits its highest values. For instance, Fig. M1 (left column) of the Supplementary information^41^ exhibits experimentally retrieved exemplary color-coded *δI*/*I* maps for the Co/Ag/Co stack with *t*_*T*_ = 10 nm and *t*_Ag_ = 0.77 nm (*H* = 1 and 0 kOe, respectively). As shown in Fig. M1^41^, fitting the experimental *δI*/*I* maps to Eq. (M.2) by using the *B*_*i*_ as adjustable parameters allows to separation of the longitudinal and transverse MOKE contributions, as well as the residual *δI*/*I* (difference between experiment and fit). This is due to the different polarization symmetries possessed by the longitudinal and transverse MOKE, thus enabling the vector magnetometry capability of the GME technique.

#### Quantification of non-uniform magnetization states via GME

Once the reflection matrix elements are obtained from the fitting routine described above, the dielectric tensor of the samples for a given magnetization state can be recovered by devising an optical model of the samples and performing a best-match model fit^43^. For an in-plane magnetized material such as the Co layers here, a dielectric tensor in the following form is usually assumed,

M.4 $$\mathop{{{{{{\boldsymbol{\varepsilon }}}}}}}\limits^{\leftrightarrow}=({\varepsilon }_{{ij}})={N}^{2}\left(\begin{array}{ccc}1 & 0 & -{iQ}{m}_{y}\\ 0 & 1 & {iQ}{m}_{x}\\ {iQ}{m}_{y} & -{iQ}{m}_{x} & 1\end{array}\right),$$

where *N* = *n* + *ik* is the refractive index and *Q* = *Q*_*r*_ + *iQ*_*i*_ the magneto-optical coupling factor, while {*m*_*x*_, *m*_*y*_} are normalized magnetization components. Eq. (M.4) takes the assumption of a uniform magnetization state, which works well, for instance, for highly epitaxial Co films^40^. However, for polycrystalline films one can expect that non-uniform (non-collinear) states of magnetization will occur within the same film, such that the off-diagonal tensor elements now take the form

M.5 $$\begin{array}{c}{\varepsilon }_{13}=-{\varepsilon }_{31}=-{iQ}\frac{\mathop{\sum}_{i}{m}_{y,i}}{G}\\ {\varepsilon }_{23}=-{\varepsilon }_{32}=-{iQ}\frac{\mathop{\sum}_{i}{m}_{x,i}}{G}\end{array},$$

with $G=\sqrt{\mathop{\sum}_{i}\left({m}_{x,i}^{2}+{m}_{y,i}^{2}\right)}$ being the number of grains in the system. During the fit process to a best-match model fit, we still treat the dielectric tensor elements as the magnetization components of a single vector resulting from the sum, such that *ε*_13_, *ε*_31_ ~ sin *γ* and *ε*_23_, *ε*_32_ ~ cos *γ*. In order to do this, however, we would need to normalize the sums of *m*_*x,i*_ and *m*_*y,i*_ as

M.6 $$\begin{array}{c}\sin \gamma=\frac{\mathop{\sum}_{i}{m}_{y,i}}{\sqrt{{\left(\mathop{\sum}_{i}{m}_{x,i}\right)}^{2}+{\left(\mathop{\sum}_{i}{m}_{y,i}\right)}^{2}}}=\frac{\mathop{\sum}_{i}{m}_{y,i}}{G^{\prime} }\\ \cos \gamma=\frac{\mathop{\sum}_{i}{m}_{x,i}}{\sqrt{{\left(\mathop{\sum}_{i}{m}_{x,i}\right)}^{2}+{\left(\mathop{\sum}_{i}{m}_{y,i}\right)}^{2}}}=\frac{\mathop{\sum}_{i}{m}_{x,i}}{G^{\prime} }\end{array},$$

where we have termed as *G*’ the quantity related to the modulus of the resulting magnetization vector. For systems formed by several magnetic moment vectors that add up, we have that

M.7 $${G^{\prime} }^{2}={\left(\mathop{\sum}\limits_{i}{m}_{x,i}\right)}^{2}+{\left(\mathop{\sum}\limits_{i}{m}_{y,i}\right)}^{2}\le \mathop{\sum}\limits_{i}\left({m}_{x,i}^{2}+{m}_{y,i}^{2}\right)={G}^{2},$$

where inequality holds for the case in which non-uniform states of magnetization are present. Thus we now define our dielectric tensor elements to be of the form

M.8 $$\begin{array}{c}{\varepsilon }_{13}=-{\varepsilon }_{31}=-i{Q}_{{{{{\rm{eff}}}}}}\sin \gamma \\ {\varepsilon }_{23}=-{\varepsilon }_{32}=-i{Q}_{{{{{\rm{eff}}}}}}\cos \gamma \end{array},$$

this brings the effect of substituting the original magneto-optical coupling factor *Q* by an effective coupling defined as

M.9 $${Q}_{{{{{\rm{eff}}}}}}=Q\frac{\sqrt{{\left(\mathop{\sum}_{i}{m}_{x,i}\right)}^{2}+{\left(\mathop{\sum}_{i}{m}_{y,i}\right)}^{2}}}{\sqrt{\mathop{\sum}_{i}\left({m}_{x,i}^{2}+{m}_{y,i}^{2}\right)}}=Q\frac{G^{\prime} }{G},$$

where the effective coupling factor *Q*_eff_ now is the original coupling factor times the relative reduction in magnetization as a result of non-uniform magnetization states. By comparing experimentally determined *Q* values at a high applied field and during reversal, we can directly estimate the reduction in magnetization as

M.10 $$\frac{M}{{M}_{0}}=\frac{{Q}_{{{{{\rm{eff}}}}}}}{{Q}_{{{{{\rm{eff}}}}},{MAX}}},$$

where *Q*_eff, MAX_ is the value of the magneto-optical coupling factor retrieved in magnetic saturation when all grains are considered to be aligned with the field and *G*′ = *G*.

### Multilayer optical model for the Co/Ag/Co samples

In order to mimic the optical, magneto-optical, as well as magnetic properties of our Co/Ag/Co multilayers in the combined optical and magnetic model, we develop a stratified optical model of our samples. A schematic of the optical model utilized is shown in the top part of Fig. M4 of^41^, for the samples with *t*_*T*_ = 10 and 15 nm. First, we chose *N* = 1.46 for the SiO_2_ overcoat, which we measured via spectroscopic ellipsometry on Si/SiO_2_ samples. Additionally, we employ the refractive index *N* = 2.4 + 4.0*i* as well as the magneto-optical coupling factor *Q* = (2.95 − 0.96*i*) × 10^−2^ for the Co layers, which we also measured for polycrystalline Co films.

Having fixed these aspects of the optical model, we can fit the Ag thickness dependence of the longitudinal parameters ${{{{\mathrm{Re}}}}}(\widetilde{\alpha })$ and ${{{{\mathrm{Re}}}}}({\widetilde{r}}_{s}\cdot {\widetilde{\alpha }}^{*})$ as well as the purely optical reflection matrix parameters ${\left|{\widetilde{r}}_{s}\right|}^{2}$ and ${{{{\mathrm{Re}}}}}({\widetilde{r}}_{s})$ in magnetic saturation (*H* > 1 kOe) by adjusting the refractive index of the of the Ag interlayer, yielding a result of *N*_Ag_ = 0.15 + 6.41*i*. The data and corresponding fits are shown in Fig. M4. It can be appreciated that while the optical model can reproduce well the linear trend of the reflection matrix parameters above 1 nm thickness, a thickness-independent approach for the refractive index of Ag does not give good results for *t*_Ag_ < 1 nm. Below this interlayer thickness, quantum mechanical interference effects most probably induce modifications in the band structure, which in turn also vary the optical properties of the material.

### Macrospin model of Co/Ag/Co stacks with a Dzyaloshinskii–Moriya type IEC

Further details of the simple macrospin model employed to understand the magnetization reversal behavior of our polycrystalline Co/Ag/Co stacks are presented (see Fig. 3 in the main manuscript text). We consider the following macrospin systems and interactions among them:

(i) Two ferromagnetic layers formed by Stoner–Wohlfarth grains with a distribution of easy axis orientations. For the simplest case considered here, each ferromagnetic layer is formed by two populations of grains, possessing anisotropy axes that are symmetric with respect to the field axis (and thus 90° away from each other). The magnetic anisotropy energy per unit area of the top (*T*) and bottom (*B*) Co layers read as
  M.11 $$\begin{array}{c}{E}_{K}^{T}=-\frac{K}{2}{t}_{T}{\cos }^{2}\left({\theta }_{1}^{T}-\frac{\pi }{4}\right)-\frac{K}{2}{t}_{T}{\cos }^{2}\left({\theta }_{2}^{T}+\frac{\pi }{4}\right)\\ {E}_{K}^{B}=-\frac{K}{2}{t}_{B}{\cos }^{2}\left({\theta }_{1}^{B}-\frac{\pi }{4}\right)-\frac{K}{2}{t}_{B}{\cos }^{2}\left({\theta }_{2}^{B}+\frac{\pi }{4}\right)\end{array},$$
  with all grains possessing the uniaxial magnetocrystalline anisotropy energy density *K*.
(ii) The grains in each Co layer interact via an intralayer exchange coupling mechanism, which regulates the inter-granular magnetization alignment within grains. For the sake of simplicity, the strength of this interaction is considered to be equal in the bottom and top Co layers. Again, by writing the energy per unit area, we have that
  M.12 $$\begin{array}{c}{E}_{J}^{T}=-J{t}_{T}\cos ({\theta }_{1}^{T}-{\theta }_{2}^{T})=-J{t}_{T}\left({s}_{1x}^{T}{s}_{2x}^{T}+{s}_{1y}^{T}{s}_{2y}^{T}\right)\\ {E}_{J}^{B}=-J{t}_{B}\cos ({\theta }_{1}^{B}-{\theta }_{2}^{B})=-J{t}_{B}\left({s}_{1x}^{B}{s}_{2x}^{B}+{s}_{1y}^{B}{s}_{2y}^{B}\right)\end{array},$$
  where *J* > 0 is the volume averaged exchange coupling energy. We have also introduced the in-plane magnetization components along the *x*- and *y*-axis for each of the spins in the Co layers, ${s}_{{ix}}^{l}=\cos {\theta }_{i}^{l}$ and ${s}_{{iy}}^{l}=\sin {\theta }_{i}^{l}$ (with *i* = 1, 2 and *l* = *T*, *B*).
(iii) The energy per unit area of the Zeeman interaction affecting the spins is
  M.13 $$\begin{array}{c}{E}_{Z}^{T}=-\frac{{M}_{0}{t}_{T}H}{2}\left(\cos {\theta }_{1}^{T}+\cos {\theta }_{2}^{T}\right)=-\frac{{M}_{0}{t}_{T}H}{2}\left({s}_{1x}^{T}+{s}_{2x}^{T}\right)\\ {E}_{Z}^{B}=-\frac{{M}_{0}{t}_{B}H}{2}\left(\cos {\theta }_{1}^{B}+\cos {\theta }_{2}^{B}\right)=-\frac{{M}_{0}{t}_{B}H}{2}\left({s}_{1x}^{B}+{s}_{2x}^{B}\right)\end{array},$$
  where *H* is the applied magnetic field along the *x*-axis and *M*_0_ represents volume averaged magnetization density.
(iv) DM type IEC between the resulting magnetization vectors of the two magnetic layers is added, favoring their perpendicular alignment. Here, we define a coupling vector $\vec{D}=D\hat{k}$ which couples the resulting magnetization vectors of the top and bottom layers via
  M.14 $${E}_{{DM}} 	=-\frac{1}{4}{{{{{\boldsymbol{D}}}}}}\cdot {\big({{{{{\boldsymbol{m}}}}}}}^{T}\times {{{{{{\boldsymbol{m}}}}}}}^{B}\big)=-\frac{D}{4}\hat{{{{{{\boldsymbol{k}}}}}}}\cdot \left({{{{{{\boldsymbol{s}}}}}}}_{1}^{T}+{{{{{{\boldsymbol{s}}}}}}}_{2}^{T}\right)\times \left({{{{{{\boldsymbol{s}}}}}}}_{1}^{B}+{{{{{{\boldsymbol{s}}}}}}}_{2}^{B}\right)\\ 	=-\frac{D}{4}\left[\left({s}_{1x}^{T}+{s}_{2x}^{T}\right)\left({s}_{1y}^{B}+{s}_{2y}^{B}\right)-\left({s}_{1x}^{B}+{s}_{2x}^{B}\right)\left({s}_{1y}^{T}+{s}_{2y}^{T}\right)\right],$$
  where the sign of the factor *D* determines the right- or left-handed helicity of the interaction, and the 1/4 factor accounts for the multiplication of 2 times 2 spins in the interaction term. This specific interaction, through the cross product of the two interacting magnetization vectors, adds to the model the two key ingredients needed to qualitatively reproduce the outcome of our experiments. On one hand it favors the perpendicular alignment of the resulting moments of the layers, giving rise to noncollinear magnetization states. On the other hand, it introduces a preferred helicity of the magnetization configuration, due to the non-commutative property of the cross product.
(v) Heisenberg type interlayer exchange interaction between the resulting magnetization vectors of the two magnetic layers.
  M.15 $${E}_{{Heis}-{IEC}}=-\frac{1}{4}{J}_{{{{{{\rm{Heis}}}}}}-{{{{{\rm{IEC}}}}}}}({{{{{{\boldsymbol{m}}}}}}}^{T}\cdot {{{{{{\boldsymbol{m}}}}}}}^{B})=-\frac{{J}_{{{{{{\rm{Heis}}}}}}-{{{{{\rm{IEC}}}}}}}}{4}\left({{{{{{\boldsymbol{s}}}}}}}_{1}^{T}+{{{{{{\boldsymbol{s}}}}}}}_{2}^{T}\right)\cdot \left({{{{{{\boldsymbol{s}}}}}}}_{1}^{B}+{{{{{{\boldsymbol{s}}}}}}}_{2}^{B}\right),$$
  where the sign of the factor *J*_Heis–IEC_ determines the FM or AFM character of the interaction,
(vi) Biquadratic interlayer exchange interaction between the resulting magnetization vectors of the two magnetic layers.

M.16 $${E}_{b}=\frac{1}{4}{J}_{b}({{{{{{\boldsymbol{m}}}}}}}^{T}\cdot {{{{{{\boldsymbol{m}}}}}}}^{B}){\,\!}^{2}=\frac{{J}_{b}}{16}{\left[\left({{{{{{\boldsymbol{s}}}}}}}_{1}^{T}+{{{{{{\boldsymbol{s}}}}}}}_{2}^{T}\right)\cdot \left({{{{{{\boldsymbol{s}}}}}}}_{1}^{B}+{{{{{{\boldsymbol{s}}}}}}}_{2}^{B}\right)\right]}^{2}$$

Thus, one can now build the total energy per unit area by summing up the contributions from the different interactions, namely

M.17 $${E}_{{TOT}}={E}_{K}^{T}+{E}_{K}^{B}+{E}_{J}^{T}+{E}_{J}^{B}+{E}_{Z}^{T}+{E}_{Z}^{B}+{E}_{{DM}}+{E}_{{{{{{\rm{Heis}}}}}}-{{{{{\rm{IEC}}}}}}}+{E}_{b},$$

where the last three terms is the only one involving interaction of spins coming from different layers. Although the present model might be very simplistic, in particular in terms of its lateral sample structure, it captures the main ingredients of the physics that is present in our Co/Ag/Co samples, constituting a first good approximation toward a better understanding of their magnetization reversal properties.

In order to solve the magnetic field dependent evolution of the magnetization configuration in this model for a given set of parameters {*K*, *J*, *D*}, we recall that the free energy of a macrospin assembly can be expressed as $F=-\mathop{\sum}_{i}{s}_{i}\cdot {\vec{H}}_{i}^{{eff}}$, summing up over all constituent spins. The effective field ${\vec{H}}_{i}^{{eff}}$ for each spin is defined as

M.18 $${\left({\vec{H}}_{i}^{{eff}}\right)}^{l}=-\frac{1}{{M}_{0}}\left(\frac{\partial {E}_{{TOT}}}{\partial {s}_{{ix}}^{l}}\hat{i}+\frac{\partial {E}_{{TOT}}}{\partial {s}_{{iy}}^{l}}\hat{j}\right),$$

where *i* = 1, 2 and *l* = *T*, *B*. From here, the metastable magnetization configuration for each applied field *H* can be obtained self-consistently by requiring each spin to be aligned with its effective field vector, hence minimizing the free energy of the system. At this point, it is also convenient to introduce the following reduced parameters:

- Ratio between bottom and top thicknesses, *r* = *t*_*B*_/*t*_*T*_
- Anisotropy field, *H*_*K*_ = 2*K*/*M*_0_
- Dimensionless applied magnetic field, *h* = *H*/*H*_*K*_ = *M*_0_ *H*/2*K*
- Reduced intralayer exchange coupling strength, *j* = *J*/2*K*
- Reduced interlayer DMI coupling strength, *d* = *D*/2*K*

In the following, we evaluate the macrospin configurations for different applied field values *h* given the dimensionless coupling strengths *j* and *d*. We also chose a bottom-to-top thickness ratio of *r* = 10 for this particular case. Fig. M5^41^ shows the field dependent evolution of the *m*_*x*_, *m*_*y*_ magnetization components of the top as well as bottom Co layers for intralayer coupling strengths *j* = 0, 0.5 and 1, as well as zero DM type interlayer coupling, *d* = 0. One of the most immediate results is the fact that the *m*_*x*_ and *m*_*y*_ vs. *h* curves are identical for the top and bottom FM layers, as they act completely independently while sharing the very same magnetic properties. One can observe that while the *m*_*x*_ component follows a field dependent hysteresis curve, the transverse component of magnetization *m*_*y*_ is zero for all field values. This is because upon lowering *h*, the spins in each layer rotate non-coherently in opposite directions. It is also worth to mention that the intralayer strength *j* controls the squareness and width of the *m*_*x*_ hysteresis loops, as it has a direct consequence on the restoring force exerted by the anisotropy axes onto the spins against the action of the applied magnetic field.

Thus for *d* = 0, there is no interaction breaking the symmetry of the system around the applied field axis (*x*-axis). The situation is different when the IEC between the magnetic layers is introduced. Fig. M6^41^ shows the field dependent magnetization evolution for a system with DM type interlayer coupling strengths of *d* = 0.04 and 0.1. One can observe here that the *m*_*x*_ components of the top and bottom layers have a very similar field dependence as for the *d* = 0 case. However, the *m*_*y*_ magnetization components of the top and bottom layer now feature a hysteretic behavior, having opposite signs with respect to each other. This means that apart from the non-coherent magnetization rotation process described before, a net coherent magnetization rotation also takes place upon lowering the field in both ferromagnetic layers. Specifically, the magnetization of the top layer deviates from the *x*-axis on the order of few degrees, while the bottom layer magnetization is tilted by a significantly smaller but still appreciable angle in the opposite direction.

This is understood in terms of the competition between the DM type interlayer coupling, promoting perpendicular alignment between the magnetization vectors of the layers, with magnetic anisotropy and intralayer interactions. While the interlayer coupling may not be capable to align both magnetizations perpendicular, the system still gains sufficient energy by partially adapting to this interaction, via deflecting the magnetizations of each layer to both sides of the applied field axis. This results into a configuration of the top and bottom layers in which the respective magnetizations are canted on the order of a few degrees, thus setting a plausible scenario for explaining our experimental observations in Co/Ag/Co films. From comparison of the *d* = 0.04 and 0.1 one can conclude that *d* determines the magnetization tilt amplitude from the *x*-axis. In addition, the ratio between the transverse magnetization components from the top and bottom layers is related by the thickness ratio, ${m}_{y}^{T}/{m}_{y}^{B}=-r$, regardless the *d* value (this being true for small *d* values and hence small tilt angles of the layer magnetizations). This is because the energy gain due to the partial fulfillment of the Zeeman or magnetic anisotropy energies is a factor of *r* = 10 times smaller for the top layer than for the bottom one, while both being equally affected by the DM type interlayer coupling despite their different thicknesses. It is worth noting that while the *m*_*y*_ components of the Co layers a show well-defined individual hysteretic behavior, the averaged transverse component of the Co/Ag/Co stack is vanishing in all cases.

Finally, the other crucial aspect to evaluate from this model is the role of the sign of *d*. One can observe that its sign defines the clockwise or counterclockwise character of the angle between the top and bottom layer magnetizations, thus defining the preferred helicity of magnetization rotation of coupled layers during reversal. Fig. M7^41^ exhibits simulated magnetization reversal curves for the cases *d* = ±0.02. As can be seen, the field dependent *m*_*y*_ values of both layers change their sign upon inverting the sign of *d*. Thus, the preferred helicity set by the DM type interlayer coupling is reflected in that the angle going from the bottom layer magnetization ***m***^*B*^ to the top layer magnetization ***m***^*T*^ is the same in the two cases, but defined to be as counterclockwise for the positive *d* case, while being clockwise for a negative *d*.

### Supplementary information

Supplementary Information

## Data Availability

The measured color-coded δI/I datasets data are available at the “Methods” section. The azimuthal dependence of the reflectivity coefficients generated in this study are provided in the Supplementary Information.

## Code availability

The codes are described in a detailed way in the “Methods” section.
