# Supplementary material for: Large interlayer Dzyaloshinskii-Moriya interactions across Ag-layers
Source: Nat Commun. 2023 Oct 30;14:6927. doi: 10.1038/s41467-023-42426-9 (PMC10616179; doi:10.1038/s41467-023-42426-9)
Supplement: Supplementary file 1 — Supplementary Information [file 41467_2023_42426_MOESM1_ESM.pdf]

SUPPLEMENTARY INFORMATION

for manuscript

"Large Interlayer Dzyaloshinskii-Moriya interactions across Ag-layers"

Jon Ander Arregi[1], Patricia Riego[1,2], Andreas Berger[1], and Elena Y. Vedmedenko[3]

[1]CIC nanoGUNE BRTA, Tolosa Hiribidea 76, E-20018 Donostia–San Sebastián, Spain

[2]Departamento de Física de la Materia Condensada, Universidad del País Vasco, UPV/EHU, E-48080 Bilbao, Spain

[3]University of Hamburg, Department of Physics, Jungiusstrasse 11a, 20355 Hamburg, Germany

I. Fractional intensity changes of the top layer.

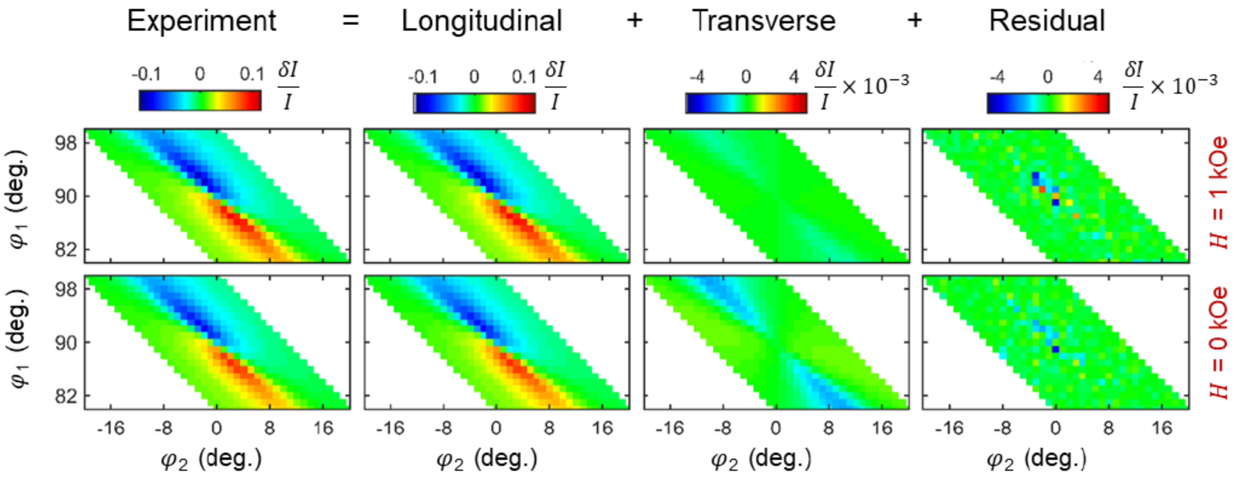

Fig. M1: **Fractional intensity changes of the top layer.** Measured color-coded $\delta I/I$ datasets as well as the corresponding separation into longitudinal and transverse MOKE contributions enabled by the fitting process. Data acquired for the t_T = 10 nm at t_{Ag} = 0.77 nm, for applied field values of H = 1 and 0 kOe (first and second row, respectively). A remarkable fit goodness of R^2 > 0.999 is obtained in both cases. A clear increase of a transverse signal is perceived in the $\delta I/I$ datasets upon reducing the field from 1 kOe to zero, as an indication of a coherent magnetization rotation process.

II. Magnetization rotation process in a polycrystalline reference Co film

We have also studied coherent and non-coherent magnetization reversal processes by means of the GME technique in a 100-nm-thick Co film sputtered onto oxidized Si substrates, under the same conditions as for the Co/Ag/Co stacks.

Fig. M2 shows experimental $\delta I/I$ maps (left column) for such a 100-nm-thick polycrystalline sample, for field values of 1 and 0 kOe (top and bottom). By following the fitting process, each map is separated into its longitudinal and transverse contribution (as well as in the residual). Compared to the data shown for the Co/Ag/Co stack, we see that no apparent transverse magnetization signal is present even in remanence. This suggests that the transverse magnetization components $m_{y,i}$ of the Co grains average out to give a total zero magnetization along this axis. Thus, the dominating reversal process consists in the non-coherent rotation of the grains, which deflect their magnetization to both sides of the applied field axis with equal probabilities during reversal. We have plotted the complete field-dependent fitting results of the experiment in Fig. M3. In particular, the left panel shows the experimentally determined reflection matrix elements $Re(\tilde{\alpha})$, $Re(\tilde{\beta})$ and $|\tilde{r}_s|^2$ during magnetization reversal (the other three parameters are not shown). The quantity $Re(\tilde{\alpha})$, proportional to the longitudinal magnetization m_x, undergoes a reduction of about 20% when going from magnetic saturation to remanence (Fig. M3(a)). On the other hand, we see that the element $Re(\tilde{\beta})$ associated with transverse MOKE is zero for the entire applied field range, meaning that no net magnetization rotation occurs in the sample during reversal (Fig. M3(b)). Finally, the reflectivity term $|\tilde{r}_s|^2$ in (Fig. M3(c)) is field-independent, as expected.

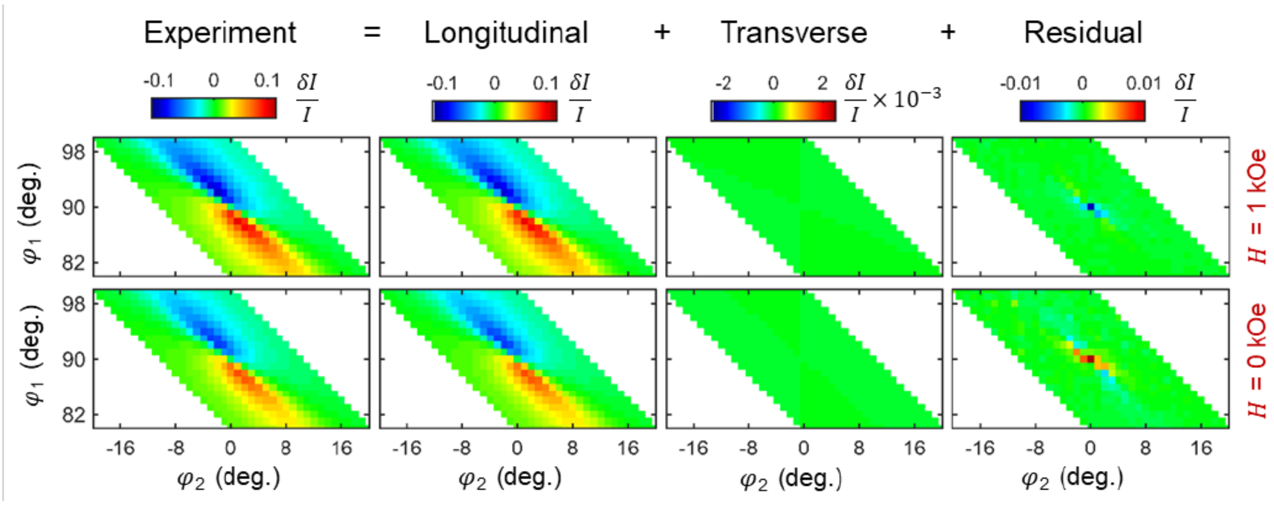

Fig. M2 Fractional intensity changes of the bottom layer. Measured color-coded $\delta I/I$ datasets as well as the corresponding separation into longitudinal and transverse MOKE contributions enabled by the fitting process. Data acquired for 100-nm-thick polycrystalline sample deposited onto oxidized

Si(001) substrates. A remarkable fit goodness of $R^2 > 0.999$ is obtained in both cases. In this case, the data suggest that there is no appreciable transverse signal even at remanence, thus coherent magnetization rotation processes being absent. We have also recovered the field-dependent dielectric tensor of the sample by performing a best-match model fit to an optical model consisting of a semi-infinite single Co layer. While the refractive index $N = n + ik$ is constant in the entire field range (Fig. M3(d)), we see that both the real and imaginary part of the magneto-optical coupling factor Q undergo a slight reduction in their absolute value as the field is lowered [Fig. M3(e)], reaching the minimum point exactly at the switching field (~ 200 Oe). Finally, the magnetization angle γ vs. H is plotted in Fig. M3(f), in which it shows a bi-stable behavior, being fully aligned along the positive field axis (~ 0º) before switching and fully aligned along the negative field axis (~ 180º) after switching. Following the description in *Section I.b* of this Methods section, we can explain this outcome in terms of the existence of non-uniform magnetization states as a result of non-coherent magnetization rotation during reversal. This process effectively lowers down the longitudinal magnetization component, and thus its associated longitudinal MOKE, while keeping the net transverse magnetization to zero, such that a zero transverse MOKE is observed, too. Thus, the decrease Q amplitude gives us a way to quantify the effective reduction of the coherently aligned magnetization vector, as it was done in the Fig. 1(b) of the manuscript, allowing us to identify non-collinear magnetization states in our samples.

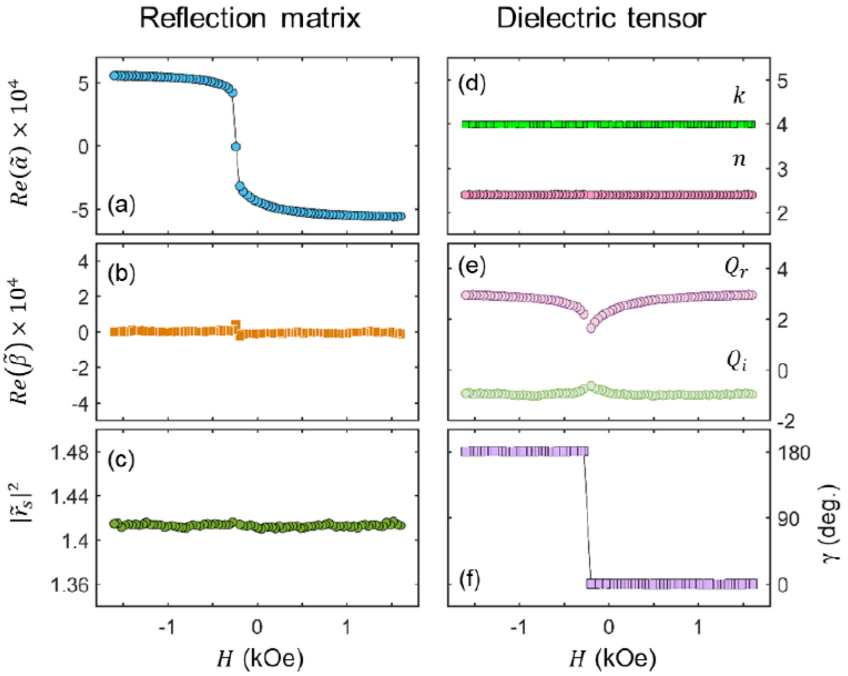

Fig. M3 Reflection matrix. Outcome of the reflection matrix fit process (left panel) and the best-match optical model fit (right panel) for GME measurement on a 100-nm-thick polycrystalline Co film.

III. Multilayer optical model for the Co/Ag/Co samples

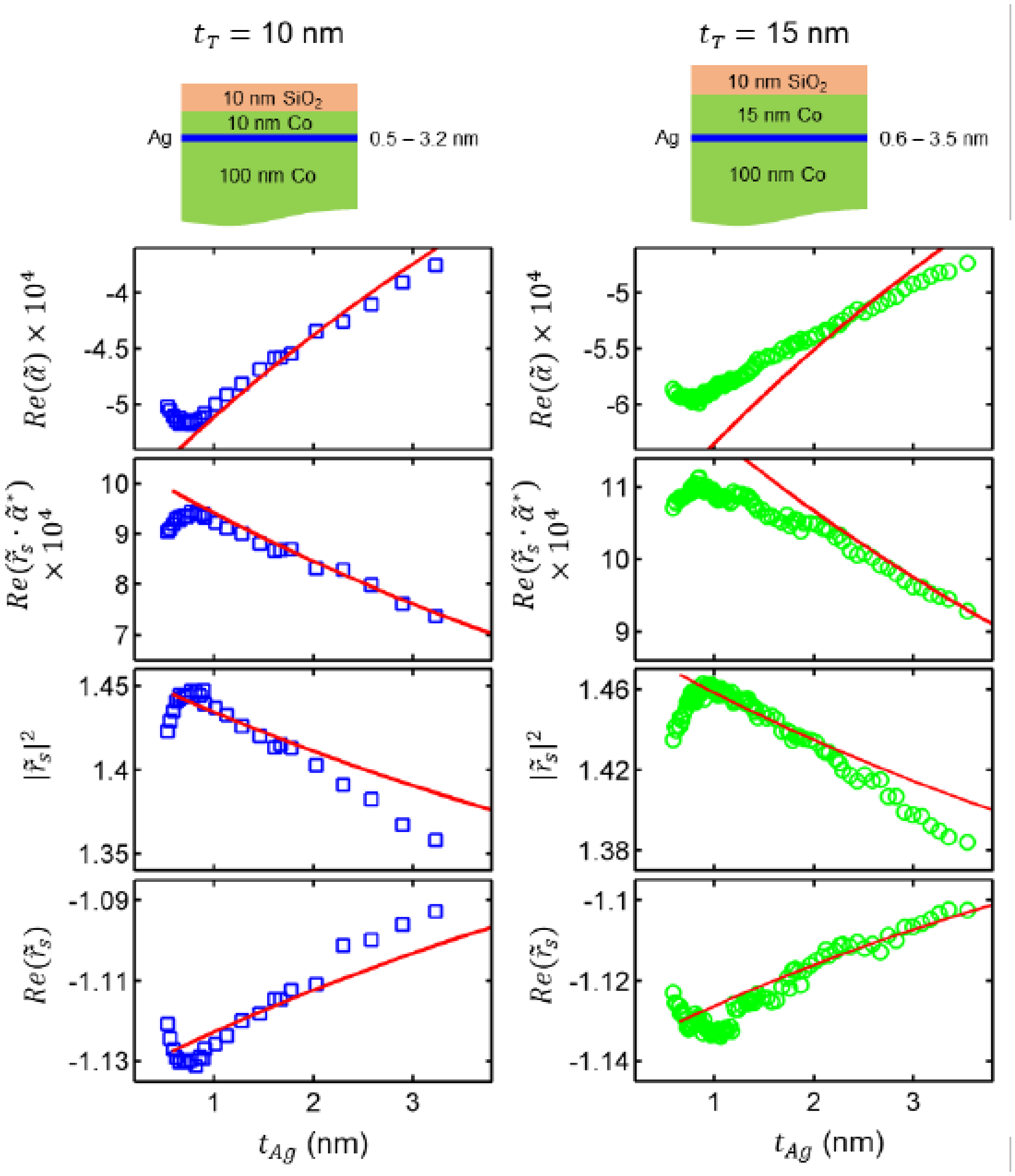

Fig. M4 Details of the optical model. (Top) Schematic of the stratified optical model built for our samples. (Bottom) Ag-thickness dependence of the longitudinal MOKE and purely optical reflectivity parameters measured in magnetic saturation ($H > 1$ kOe) for the samples with t_T = 10 and 15 nm. The red curves contitute fits of the measured data by adjusting the refractive index of the Ag interlayer.

IV. Macrospin model of Co/Ag/Co stacks with a Dzyaloshinskii-Moriya type interlayer exchange coupling (see description in Methods)

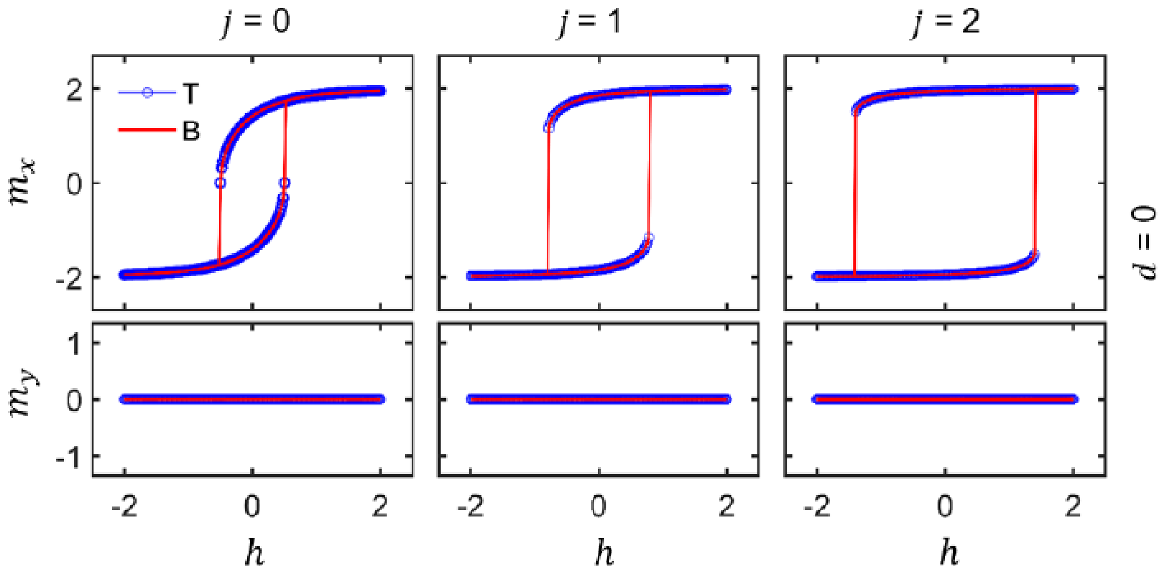

Fig. M5 Modelled magnetization curves for varying intralayer DMI. Field dependence of the magnetization components m_x and m_y for different strengths intralayer coupling strength values j and zero effective interlayer coupling, d = 0, $J_{\text{Heis-IEC}} = 0, J_b = 0$.

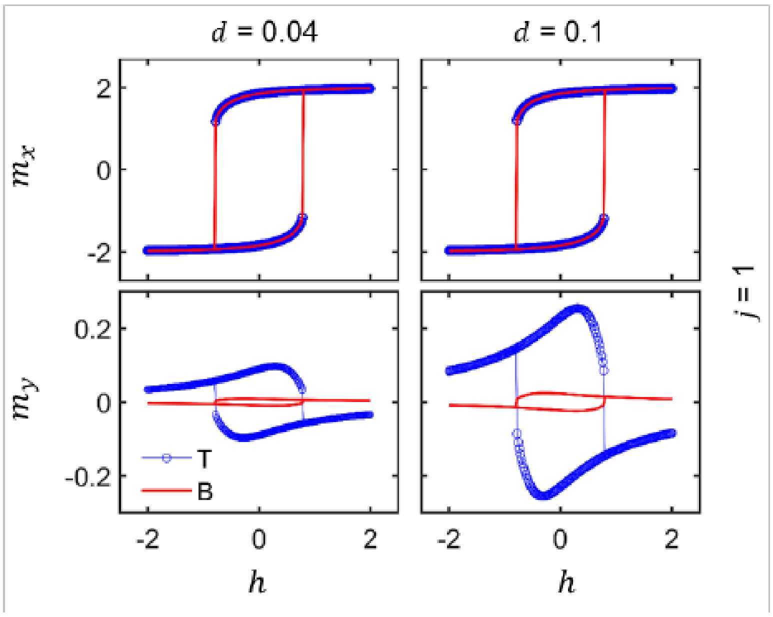

Fig. M6 Modelled magnetization curves for varying intralayer exchange interaction. Field dependence of the magnetization components m_x and m_y for different DM type interlayer coupling strengths d and j = 1 and $J_{\text{Heis-IEC}} = 0, J_{\text{b}} = 0$.

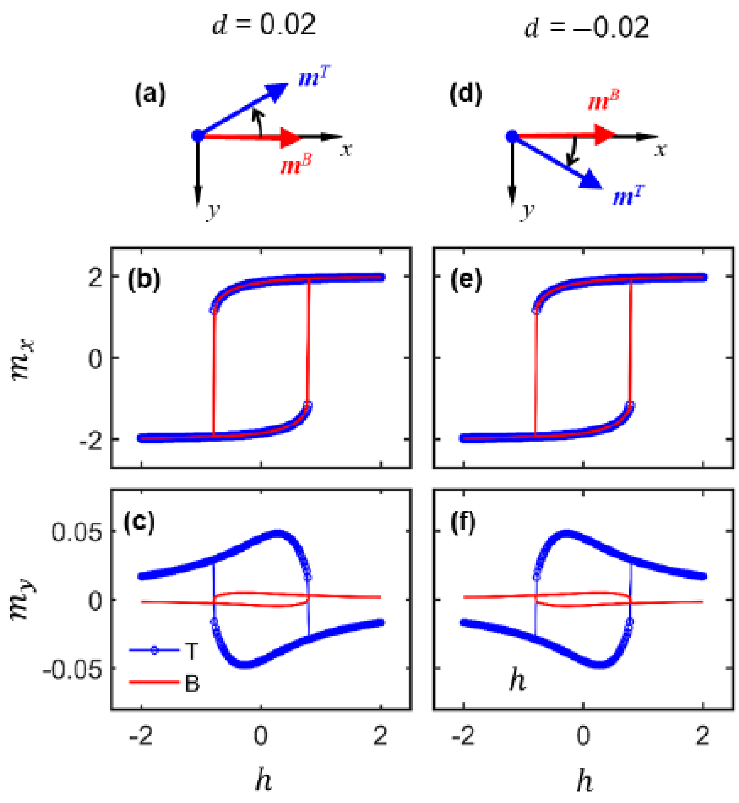

Fig. M7 Comparison of magnetization curves for opposite orientation of the intralayer DMI. (a) Decreasing field branch magnetization configuration at remanence for the top and bottom magnetization vectors $\boldsymbol{m}^T$ and $\boldsymbol{m}^B$, for coupling strengths j = 1, d = 0.02 . (b), (c) show the field dependent evolution of the magnetization components for the same case. (d) – (f) display the same schematics and simulated data for a system with j = 1, d = –0.02 and $J_{Heis-IEC} = 0, J_b = 0$.

The angles of the magnetization vectors in the schematics (a) and (d) are multiplied by 10 for clarity.

V. Comparison of Heisenberg type, biquadratic and Dzyaloshinsky-Moriya interlayer energy contributions.

The interlayer exchange interaction between bottom and top layers in a magnetic trilayer has three main contributions $E_{IEC} = -\mathrm{J}_{\mathrm{IEC-Heis}}\, \boldsymbol{M}_{\mathrm{t}} \cdot \boldsymbol{M}_{\mathrm{b}} - \mathbf{D}_{\mathbf{IL-DMI}}\, \boldsymbol{M}_{\mathrm{t}} \times \boldsymbol{M}_{\mathrm{b}} + \mathrm{J}_{\mathrm{b}}\, (\boldsymbol{M}_{\mathrm{t}} \cdot \boldsymbol{M}_{\mathrm{b}})^2$ as described in the introduction. For Co/Ag multilayers only the Heisenberg type AF interlayer coupling was measured to be antiferromagnetic $\mathrm{J}_{\mathrm{IEC-Heis}} = -0.014\ \mathrm{mJ/m^2}$[33]. While the combination of Heisenberg type AF interlayer coupling ($J_{\text{ICE-Heis}}$<0) and a biquadratic interlayer coupling leads the non-collinear orientation of the magnetization in two layers at a given angle Δ_{min}, there are always two equivalent

energy minima for the $+\Delta_{min}$ and $-\Delta_{min}$ (the blue curve in the Fig. S8). When the IL-DMI is included (black curve in Fig. S1), the energies corresponding to $+\Delta_{min}$ and $-\Delta_{min}$ become not equivalent. The system became not simply noncollinear, but rather chiral.

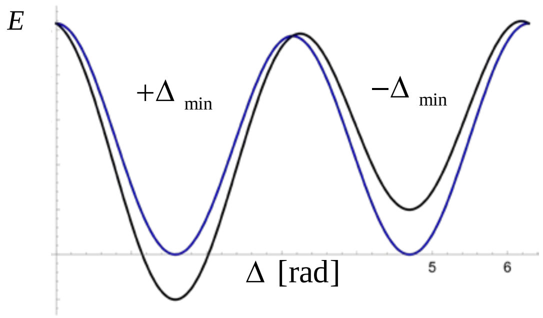

Fig. S1: Comparison of the energy landscape if Heisenberg type $-J_{IEC-Heis} = \boldsymbol{M}_t \cdot \boldsymbol{M}_b$, ($\boldsymbol{J_{IEC-Heis}} = -0.014\, mJ/m^2$) and biquadratic exchange interactions $E_b = J_b\, (\boldsymbol{M}_t \cdot \boldsymbol{M}_b)^2 (J_b = 0.075\, mJ/m^2)$ are present (blue curve) with the complete IEC including IL-DMI (black $E_{IL-DMI} = -\boldsymbol{D}_{IL-DMI}\, \boldsymbol{M}_t \times \boldsymbol{M}_b$, $D_{IL-DMI} = 0.1\, mJ/m^2$) for zero field and zero anisotropy. The two energy minima of the blue curve are equivalent. Inclusion of the IL-DMI breaks the chiral symmetry and one sense of rotation is favorized.

Experimentally, our reflectivity coefficient show that left- and right rotation is strongly not equivalent. Given that every experimental GME map consists of 441 individual hysteresis loops, the stochastic nature of equivalent non-chiral minima should have led to an averaging out of any effect that is not chiral in nature. On the same sample, we find that the helicity changes when the interlayer thickness changes, even though everything else in the experiment is identical. So, an accidental bias into one helicity by an imperfect experiment can be excluded.

In the following we perform quantitative analysis of $J_{IEC-Heis}$ and J_b. Fig. S2 left shows pure $J_{IEC-Heis} = -0.014$ mJ/m^2 (red curve), pure D_{IL-DMI}=-0.172 mJ/m^2 (blue curve, the value is taken from our measurements at t_{Ag}=0.77 nm and t_{Co}=10 nm), and the sum of both terms (black curve). The energy minimum of the curve including $J_{IEC-Heis}$ is shifted by less than 1°. These values lie within standard deviation of our measurements.

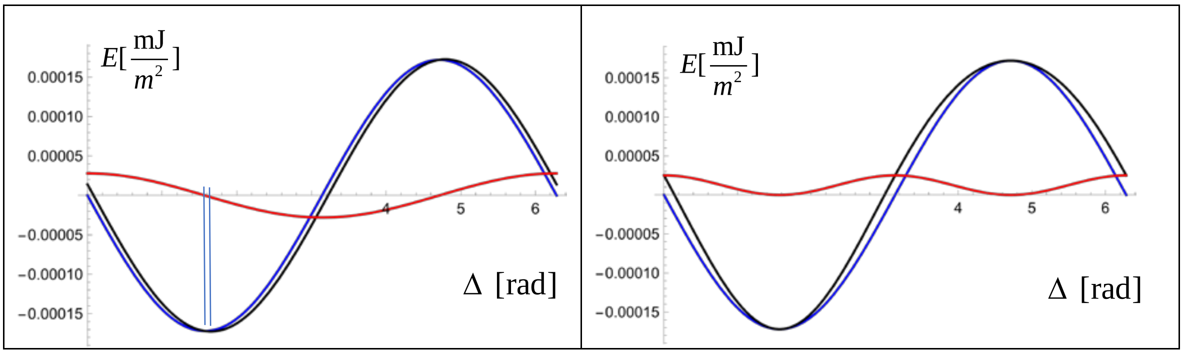

Fig. S2: (left) Heisenberg type interlayer exchange interaction energy $E_{IEC-Heis} = -J_{IEC-Heis}\, M_t \cdot M_b$, ($J_{IEC-Heis} = -0.014\, mJ/m^2, red\ curve$), IL-DMI (black $E_{IL-DMI} = -\boldsymbol{D}_{IL-DMI}\, \boldsymbol{M}_t \times \boldsymbol{M}_b$, $D_{IL-DMI} = 0.172\, mJ/m^2$) $(blue\ curve)$ and their sum (black curve); (right) biquadratic exchange interaction $E_b = J_b\, (M_t \cdot M_b)^2 (J_b = 0.025\, mJ/m^2)(red\ curve)$, IL-DMI (black $E_{IL-DMI} = -\boldsymbol{D}_{IL-DMI}\, \boldsymbol{M}_t \times \boldsymbol{M}_b$, $D_{IL-DMI} = 0.172\, mJ/m^2$) $(blue\ curve)$ as well as their sum as a function of relative angle Δ between the net magnetizations of top $\boldsymbol{M}_t$ and bottom $\boldsymbol{M}_b$ Co layers (black curve) at zero field.

Fig. S2 right shows pure $J_b = -0.025\ mJ/m^2$ (red curve), pure IL-DMI=-0.172 mJ/m^2 (blue curve), and the sum of both terms (black curve). The energy minimum at remanence does not shift

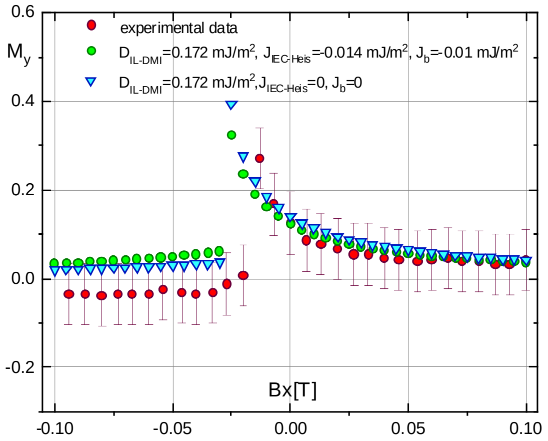

Fig. S3: An example of best fits of field dependent experimental data (at t_{Ag}=0.77 nm and t_{Co}=10 nm) from the main manuscript fitted by a model including an effective IL-DMI interlayer coupling only and by the extended model including Heisenberg type IEC and biquadratic IEC.

independently on the strength of the biquadratic interaction. Thus, the biquadratic interaction will not influence the remanence equilibrium magnetization. Only the shape of magnetization curve can be affected.

The fit of experimental data using all three energy parameters appears not possible, because the corresponding curves lie within the standard error of our fit procedure and polycrystalline nature of

our samples as can be seen in Fig. S3. However, one thing is absolutely clear: the experimental data can only be explained, if an effective IL-DMI interlayer coupling is present.

Both curves lie within the standard deviation margins of the experiment. So, we cannot distinguish between them. The values of $\mathrm{J_b}$ and $\mathrm{J_{IEC-Heis}}$ are order of magnitude weaker than that of $D_{\mathrm{IL-DMI}}$. From these data we conclude that we are not able determine other than IL-DMI interactions reliably and limit ourselves to determination of the strongest effective chiral contribution.

VI. Combined optical and magnetic model fit results for t_T = 15 nm

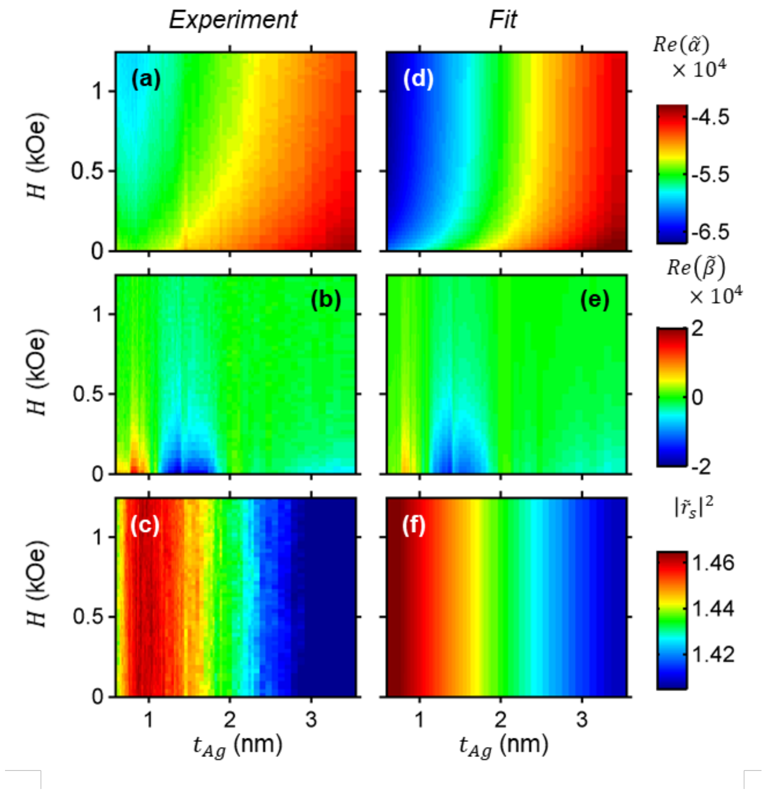

Fig. S4:

Color-coded maps of the experimentally determined reflection matrix quantities (a) $Re(\tilde{\alpha})$, (b) $Re(\tilde{\beta})$, and (c) $|\tilde{r}_s|^2$ vs. t_{Ag}, H for the sample with t_T = 15 nm. (d)-(f) show the side-by-side corresponding model fits according to the combined optical and magnetic model.

VII. Azimuthal dependence of the reflectivity coefficients for t_T = 15 nm

In order to verify that the observed IL-DMI and associated magnetization rotations are indeed caused by the helicity of the interlayer coupling, we have measured the resulting magnetization orientation at different field values for different sample orientations. Hereby, the experimental range of sample orientations that we could explore was limited, given that we had to rotate our elongated samples in the narrow gap of the electromagnet. Thus, only an orientation range of η from - 4° to + 4° was accessible, with η being the physical rotation angle away from the proper sample alignment, in which the field is applied perpendicular to the major sample axis, as shown in Fig. S5(a). The total rotation range of 8° that we covered in our experiment is very meaningful, given that it is of the same order of

magnitude as the zero-field magnetization rotation away from the field axis. If the sample has a 6°/10° easy axis rotation and that is the reason, why we see a 6°/10° magnetization rotation under the normal measurement geometry in remanence, this magnetization rotation should change to 2°/6° is we rotate our sample by 4° into the direction of the easy axis and it should change to 10°/14°, if we rotate 4° against it. So, our $\pm$ 4 ° rotation range should lead to a 2° - 14° rotation variation, which it does not. We do not observe any significant changes in measured coefficients. Thus, the total rotation range of 8° that we covered in our experiment is very meaningful, given that it is of the same order of magnitude as the zero-field magnetization rotation away from the field axis.

Figure S5(b) shows the corresponding experimental results of the transverse MOKE amplitude $Re(\tilde{\beta})$ for 3 different field values, namely H = 0 Oe (red squares), H = 250 Oe (green squares) and H = 1250 Oe (blue squares), measured for the t_T = 15 nm sample at the t_{Ag} = 1.35 nm interlayer thickness position. The data show that the transverse effect is always present in low fields and that the magnetization direction can be rotated into the longitudinal configuration for all sample orientations. More importantly, we find that there is no relevant sample orientation dependence that is observed in our data, exactly as one would expect for an IL-DMI effect that should not depend on the azimuthal sample orientation.

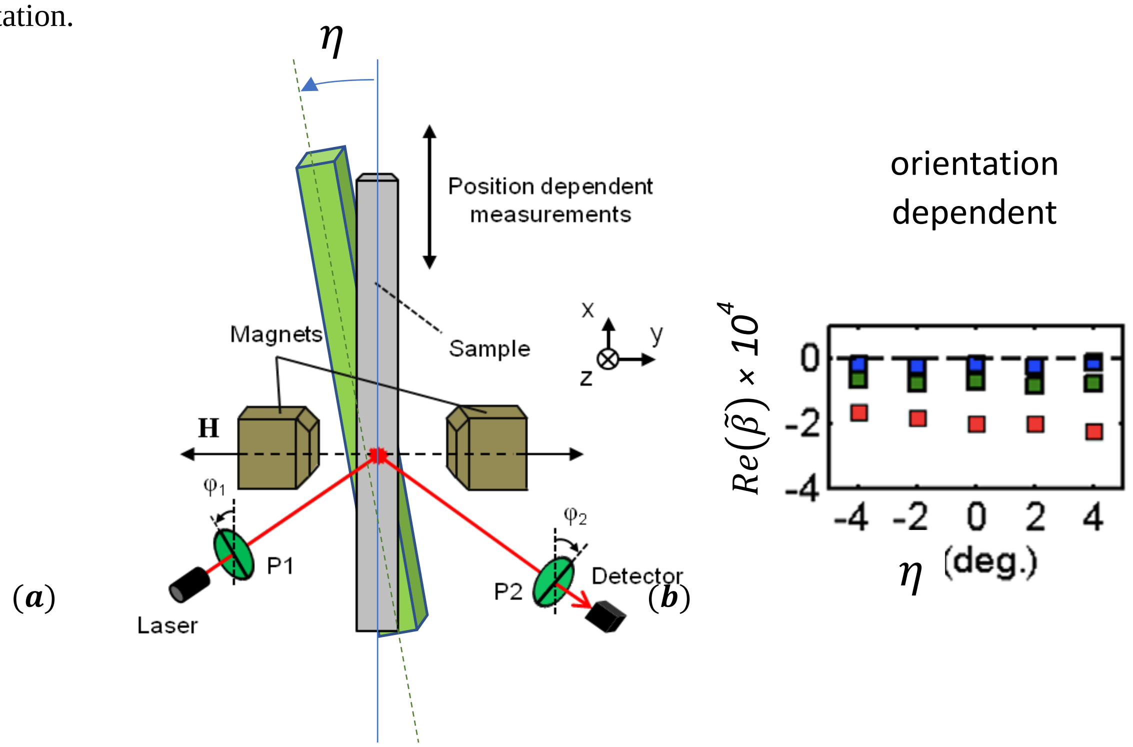

Fig. S5: (a) Schematic representation of the experimental design for the measurement of the azimuthal dependence of the reflectivity coefficients. (b) Azimuthal orientation dependence of the transverse magneto-optical Kerr effect $Re(\tilde{\beta})$, which is proportional to the magnetization rotation away from the applied field direction. The data correspond to H = 0 Oe (red squares), H = 250 Oe (green squares) and H = 1250 Oe (blue squares), measured for the t_T = 15 nm sample at the t_{Ag} = 1.35 nm interlayer thickness position.
